# Improving the Thermostability and Optimal Temperature of a Lipase from the Hyperthermophilic Archaeon *Pyrococcus furiosus* by Covalent Immobilization

**DOI:** 10.1155/2015/250532

**Published:** 2015-03-08

**Authors:** Roberta V. Branco, Melissa L. E. Gutarra, Jose M. Guisan, Denise M. G. Freire, Rodrigo V. Almeida, Jose M. Palomo

**Affiliations:** ^1^Programa de Pós-Graduação em Bioquímica, Instituto de Química, Universidade Federal do Rio de Janeiro, Avenida Athos da Silveira Ramos 149, Block A, 5th Floor, Room 541, 21941-909 Rio de Janeiro, RJ, Brazil; ^2^Departamento de Biocatálisis, Instituto de Catálisis (CSIC), Campus UAM, Cantoblanco, 28049 Madrid, Spain; ^3^Departamento de Engenharia Bioquímica, Escola de Química, Universidade Federal do Rio de Janeiro, Avenida Athos da Silveira Ramos 149, Block E, 2nd Floor, Room 203, 21949-909 Rio de Janeiro, RJ, Brazil

## Abstract

A recombinant thermostable lipase (Pf2001Δ60) from the hyperthermophilic Archaeon *Pyrococcus furiosus* (PFUL) was immobilized by hydrophobic interaction on octyl-agarose (octyl PFUL) and by covalent bond on aldehyde activated-agarose in the presence of DTT at pH = 7.0 (one-point covalent attachment) (glyoxyl-DTT PFUL) and on glyoxyl-agarose at pH 10.2 (multipoint covalent attachment) (glyoxyl PFUL). The enzyme's properties, such as optimal temperature and pH, thermostability, and selectivity, were improved by covalent immobilization. The highest enzyme stability at 70°C for 48 h incubation was achieved for glyoxyl PFUL (around 82% of residual activity), whereas glyoxyl-DTT PFUL maintained around 69% activity, followed by octyl PFUL (27% remaining activity). Immobilization on glyoxyl-agarose improved the optimal temperature to 90°C, while the optimal temperature of octyl PFUL was 70°C. Also, very significant changes in activity with different substrates were found. In general, the covalent bond derivatives were more active than octyl PFUL. The *E* value also depended substantially on the derivative and the conditions used. It was observed that the reaction of glyoxyl-DTT PFUL using methyl mandelate as a substrate at pH 7 presented the best results for enantioselectivity (*E* = 22) and enantiomeric excess (ee (%) = 91).

## 1. Introduction

Lipases (EC 3.1.1.3) and esterases (EC 3.1.1.1) are enzymes classified as ester hydrolases that catalyze the hydrolysis of ester bonds under aqueous conditions. However, in nonaqueous media, these enzymes catalyze the reverse reaction, esterification. These enzymes can also resolve racemic mixtures with high enantio- and regioselectivity, producing intermediates of interest for organic synthesis [1–5].

One of the most widely used means of solving the problem of enzyme stability is enzyme immobilization [6]. Lipases and esterases have been immobilized using a variety of immobilization processes, such as entrapment, covalent, ionic bonding, and physical adsorption on porous supports. The use of immobilized enzymes offers significant advantages in industrial processes over soluble enzymes, such as the ease of recovery of the biocatalyst and products; continuous processing, prevention of the formation of aggregates in organic media, reduction of denaturing effects, and changing of physicochemical properties [7–10]. Another way to minimize the instability of these biocatalysts is to select the enzymes produced by thermophilic microorganisms, since they are more thermostable and resistant to the action of organic solvents than mesophilic enzymes. For this reason, they are regarded as particularly promising biocatalysts for biotechnological usage [11–14].

Almeida et al. [15] cloned and expressed the orf PF2001 from* Pyrococcus furiosus* fused to Thioredoxin (TRX) in* Escherichia coli.* The authors characterized the enzyme and classified it as an esterase. This enzyme shows optimal activity at 60°C and pH 7.0, with 90% stability at 75°C for 120 minutes in the presence of Triton X-100. When immobilized on different hydrophobic supports, such as polypropylene (MP Accurel 1000), butyl Sepabeads and octadecyl Sepabeads showed 240%, 140%, and 237% hyperactivation, respectively [16, 17]. Furthermore, the assessment of storage stability showed that the enzyme immobilized on octadecyl Sepabeads retained 100% residual activity after 30 days of storage. In view of its immobilization at low ionic strength and its hyperactivation characteristics, the author reclassified this enzyme as a lipase. Alquéres et al. [18] purified the lipase fused to thioredoxin, cleaved this tag using enterokinase, and characterized the enzyme with and without the fusion protein in the presence and absence of Triton X-100. The authors found no influence of thioredoxin on optimal temperature, but the absence of Triton X-100 increased the optimal temperature of this enzyme to 80°C. The temperature stability and hyperactivation data show great potential for biotechnological applications for this enzyme.

**Scheme 1:**
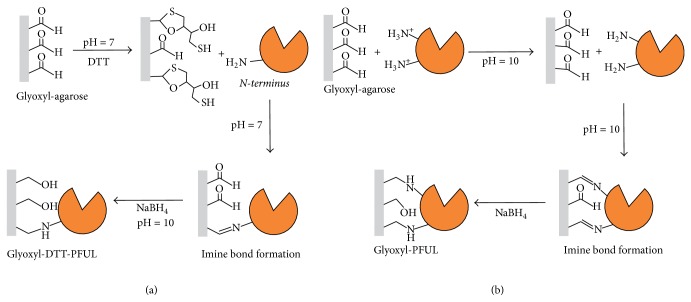
Immobilization of PFUL by different strategies: (a) in glyoxyl-DTT agarose; (b) in glyoxyl agarose.

**Scheme 2:**
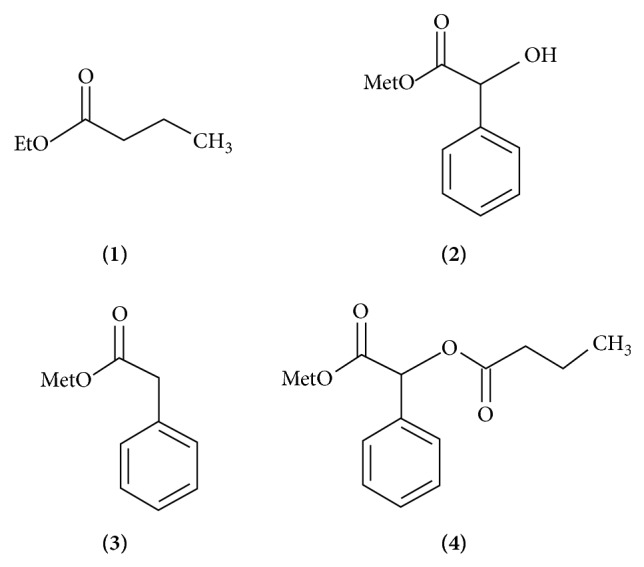
Different esters hydrolyzed by immobilized PFUL preparations. Ethyl butyrate** (** **1)**, (R,S)-methyl mandelate** (** **2)**, phenylacetic acid methyl ester** (** **3)**, (R,S)-2-O-butyryl-2-phenylacetic acid** (** **4)**.

This work combines the application of a thermophilic lipase from* Pyrococcus furiosus* (PFUL) with different immobilization strategies in order to obtain a more stable biocatalyst with promising catalytic properties. Hydrophobic immobilization was carried out on octyl-agarose. Furthermore covalent immobilization on different supports was performed, providing enzymes immobilized through one-point (glyoxyl-DTT PFUL) and multipoint (glyoxyl PFUL) covalent bonds (Scheme 1). Temperature and pH were determined using a response surface methodology. Stability at high temperature and activity using different substrates (ethyl butyrate** (** **1)**, (R,S)-methyl mandelate** (** **2)**, phenylacetic acid methyl ester** (** **3)**, (R,S)-2-O-butyryl-2-phenylacetic acid** (** **4)**) and enantioselectivity based on the kinetic resolution of** (** **2)** and** (** **4)** (Scheme 2) were done.

## 2. Materials and Methods

### 2.1. Supports and Reagents

Octyl-agarose was purchased from Pharmacia Biotech (Uppsala, Sweden) and Agarose 10 BCL was from Agarose Bead Technologies. Glyoxyl-agarose was prepared as previously described [19]. Ethyl butyrate** (** **1)**, (R,S)-methyl mandelate** (** **2)**, phenylacetic acid methyl ester** (** **3)**, (R,S)-2-O-butyryl-2-phenylacetic acid** (** **4)**, p-nitrophenylbutyrate (pNPB), Triton X-100, dithiothreitol (DTT), and sodium borohydride were purchased from Sigma Chem. Co. Other reagents were of analytical grade.

### 2.2. Enzyme Production

The production of the lipase Pf2001Δ60 (PFUL) was carried out as described by Almeida et al. [15] with minor modifications.* E. coli* BL21 (DE3) Rosetta transformed with the plasmid pET25PF2001Δ60, kindly donated by Dr. Nathalia Varejão Nogueira da Paz (IBqM – UFRJ), was grown in LB broth (0.5% yeast extract, 1% tryptone and 0.5% NaCl) containing ampicillin (100 *μ*g/L) and chloramphenicol (12.5 *μ*g/L) at 35°C and 200 rpm, until Abs_600 nm_ = 0.3, when it was induced with 0.5 mM IPTG and further incubated for 3 hours. The cells were centrifuged and stored at −20°C until their use. The enzyme extract was obtained through the resuspension of the cells in sodium phosphate buffer (50 mM, pH 7.0) and later disruption by sonication (until the crude extract was observed to be homogeneous). The crude extract was centrifuged (11,000 g, 4°C, 5 minutes) and the supernatant was lyophilized and stored at 20°C for use in the immobilization experiments.

### 2.3. Activity Determination

The activity of the supernatant and enzyme immobilized suspension were analyzed spectrophotometrically measuring the rise in absorbance at 348 nm produced by the release of p-nitrophenol (pNP) in the hydrolysis of 0.4 mM pNPB in 25 mM sodium phosphate at pH 7. The assays were determined at 65°C (*ϵ* = 3.5202 M^−1^ cm^−1^) and to initialize the reaction, 0.05–0.2 mL lipase solution or suspension was added to 2.5 mL substrate solution with magnetic stirring. An international unit of p-nitrophenol was defined as the amount of enzyme necessary to hydrolyze one *μ*mol of pNPB/min under the conditions described above [20].

The activity assays for the experimental design analysis (Section 2.8) were carried out using 4-methylumbelliferyl heptanoate (MUF-Hep) as substrate in a Varian Cary Eclipse spectrofluorimeter, as described elsewhere [17]. The immobilized enzyme (2.0 mg) was added to 3.3 mL reaction mixture (0.1% gum arabic in a 50 mM sodium phosphate buffer) with magnetic stirring (200 rpm) at diferent temperatures and pHs according to matrix of experimental design (Table 1). 12 *μ*L MUF-Hep (25 mM in ethylene glycol monomethyl ether) was added to start the reaction. The progress of the reaction was evaluated measuring the increase of fluorescence emission (*λ*_ex_ = 323 nm and *λ*_em_ = 448 nm) due to the release of MUF. All rates were measured during the linear part of the progress curve. A standard curve was constructed with 4-methylumbelliferone (MUF). One unit of activity was defined as the amount of enzyme required to release 1 *μ*mol of MUF per minute under the conditions described above.

### 2.4. Immobilization on Octyl-Agarose and Purification of the Enzyme

The recombinant enzyme was purified from crude extract obtained as Section 2.2 by interfacial adsorption, as previously described [20].

The enzyme was diluted in 50 mL 25 mM phosphate buffer, pH 7 (up to a final concentration of 0.147 mg of protein/mL), and the enzyme solution was added to 6 g octyl-agarose. The reaction was maintained for 3 h under slight agitation. After that, the suspension was vacuum-filtered and the solid was washed several times with distilled water. The immobilization process was monitored by determining the enzyme activity present in the supernatant over time. Immobilization efficiency is an important parameter and must be determined, showing the amount of lipase that has been adsorbed by the support, in other words, the amount of enzyme which was removed from the immobilization solution. This parameter can be calculated by (1) and 1a below:

(1) $$\begin{array}{c} E\left(\%\right)=\frac{U_{\text{theor}}}{U_{\text{input}}}\cdot 100, \end{array}$$

$$\begin{array}{cc} \left(1\text{a}\right) & U_{\text{theor}}=U_{\text{input}}-U_{\text{output}}, \end{array}$$

where *U*_theor_ is the units of activity adsorbed on the support, calculated by 1a; *U*_input_ is the units of enzyme available for immobilization; *U*_output_ is the units of activity remaining after the immobilization process.

Hyperactivation was evaluated by activity retention, comparing theoretical adsorbed activity (difference between the activity in the supernatant at the beginning and end of the immobilization process, expressed as U/g support) with the experimentally determined activity of the immobilized enzyme. Hyperactivation is when the value exceeds 100%. Activity retention *R*(%) was calculated by

(2) $$\begin{array}{c} R\left(\%\right)=\frac{U_{\text{imo}}}{U_{\text{theor}}}\cdot 100, \end{array}$$

$$\begin{array}{cc} \left(2\text{a}\right) & U_{\text{theor}}=U_{\text{input}}-U_{\text{output}}. \end{array}$$

For the preparation of the covalent immobilized catalysts, the lipase was desorbed from the support (1 g) by adding 10 mL of a solution of 25 mM phosphate buffer, pH 7, with 0.3% Triton X-100 (v/v), incubated for 1 h until desorption was complete. SDS-PAGE gel revealed just one protein band. A final solution of 94 *μ*g purified lipase/mL was obtained.

### 2.5. One-Point Covalent Immobilization of PFUL

1 g glyoxyl-agarose support was added to 10 mL purified PFUL solution (94 *μ*g_lip_/mL) with 50 mM DTT. The suspension was then stirred for 3 h at pH 7 and 25°C. Periodically, samples of the supernatant and suspension were withdrawn, and enzyme activity was measured as described above. Finally, the pH was adjusted to 10 and preparations were reduced by the addition of 10 mg sodium borohydride for 30 min, filtrated, and then washed with water. This immobilization process is represented in Scheme 1(a).

### 2.6. Immobilization of PFUL by Multipoint Covalent Attachment

The pH of 10 mL purified PFUL solution was adjusted to 10.2. Then, 1 g glyoxyl-agarose support was added. The suspension was stirred for 19 h at 25°C. Periodically, samples of the supernatant and suspension were withdrawn, and enzyme activity was measured as described above. Finally, the preparations were reduced by the addition of 10 mg sodium borohydride for 30 minutes, filtrated, and then washed with water. This immobilization process is represented in Scheme 1(b).

### 2.7. Thermostability

The biocatalysts (0.3 g) were incubated in 2 mL 25 mM sodium phosphate buffer at 70°C, pH 7, for 48 h. The remaining activity at different times was measured by the assay described above using pNPB as a substrate [21].

### 2.8. Optimal Temperature and pH

Full factorial experimental design (3^2^) was used to characterize the enzyme immobilized on octyl and glyoxyl agarose. Two variables were studied: pH (from 6 to 8) and temperature (from 50 to 90°C). Table 1 shows real and encoded pH and temperature values for each assay. The results were analyzed by statistical experimental tools (software Statistica 7) and a model was created to describe the lipase activity obtained as a function of pH and temperature. All terms were included in the model.

### 2.9. Enzymatic Hydrolysis of Different Esters

The hydrolysis of** (** **1)** was performed by adding 0.2 g immobilized enzyme to 4 mL 10 mM of substrate in 25 mM sodium phosphate buffer at pH 7 and 25°C. The hydrolysis of** (** **2)** was performed by adding 0.2 g immobilized enzyme to 1 mL of 5 mM substrate in 25 mM sodium phosphate buffer at pH 7, or 10 mM buffer sodium acetate at pH 5, both at 25°C. The hydrolysis of** (** **3)** was performed by adding 0.2 g immobilized enzyme to 2 mL of 5 mM substrate in 25 mM buffer sodium phosphate at pH 7 and 25°C. The hydrolysis of** (** **4)** was performed by adding 0.2 g immobilized enzyme to 1 mL of 0.5 mM substrate in 25 mM sodium phosphate buffer at pH 7, or 1 mL of 0.5 mM substrate in 10 mM sodium acetate buffer at pH 5, both at 25°C. The substrates** (** **1–4)** can be observed in Scheme 2.

The degree of hydrolysis of** (** **1–4)** was analyzed by reverse-phase HPLC (Spectra Physics SP 100 coupled to a Spectra Physics SP 8450 UV detector) on a Kromasil C18 column (15 × 0.4 cm) supplied by Analisis Vinicos (Spain). Each assay was performed at least in triplicate. Elution was performed with an acetonitrile mobile phase (30%, v/v) and 10 mM ammonium phosphate (70%, v/v) at pH 2.95. The flow rate was 1 mL/min. Elution was monitored by recording absorbance at 225 nm.

### 2.10. Determination of Enantiomeric Excess

The enantiomeric excess (ee) of the produced acid (mandelic acid) was analyzed by reversed-phase chiral HPLC. The column was a Chiracel OD-R and the mobile phase was an isocratic solution of (5%, v/v) acetonitrile and (95%, v/v) 0.5 M NaClO_4_/HClO_4_ at pH 2.3. The analyses were performed at a flow of 0.5 mL/min by recording absorbance at 225 nm.

### 2.11. Calculation of *E*-Value

The enantiomeric ratio (*E*) was defined as the ratio between the percentage of hydrolyzed R and S isomers (from racemic mixture) at 10% and 20% hydrolysis with first-order reaction kinetics. R- and S-isomers were used as standard enantiomerically pure products. Also, the *E* value was calculated from the enantiomeric excess of the product acid (ee_*p*_) and the conversion degree (*c*) using (3), described by Chen et al. [22]:

(3) $$\begin{array}{c} E=\frac{\ln\left[1-c\left(1+\text{e}{\text{e}}_{p}\right)\right]}{\ln\left[1-c\left(1-\text{e}{\text{e}}_{p}\right)\right]}. \end{array}$$

## 3. Results and Discussion

### 3.1. Immobilization of PFUL by Different Strategies

The lipase of* P. furiosus* was immobilized by hydrophobic interaction on octyl-agarose and by covalent bond on glyoxyl-DTT-agarose and glyoxyl-agarose. On octyl-agarose 85% of initial activity was immobilized on the support (yield) and 132% hyperactivation was attained. The enzyme immobilized on covalent supports (Glyoxyl-DTT-agarose and Glyoxyl-agarose) showed 66% and 44% immobilization yields, respectively, and these biocatalysts showed retention activity of 70% and 96%, respectively.

### 3.2. Thermostability

**Figure 1:**
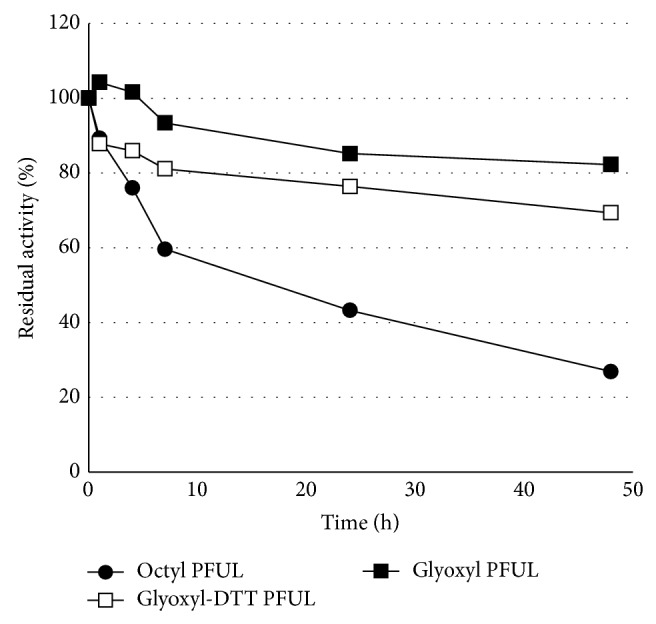
Stability at 70°C of the enzyme immobilized on different supports (hydrophobic supports and covalent bonding). The biocatalysts were incubated at different times and the residual activities were measured using pNPB as substrate.

The stability of the different immobilized preparations at different temperatures was studied to evaluate the scope of this methodology.  Figure 1 illustrates the thermostability of the enzyme immobilized on different supports. The highest enzyme stability after 48 h incubation at 70°C was achieved with immobilization on Glyoxyl-agarose (82%), whereas the enzyme immobilized on this support in the presence of DTT at pH 7 maintained around 69% activity (multipoint and one-point covalent bonding, resp.). Surprisingly, the immobilization of the enzyme on octyl-agarose (interfacially activated), which is generally the best way to stabilize lipases, based on active conformation stabilization [23, 24], yielded the worst lipase biocatalyst (only 27% remaining activity). The thermostability results for the enzyme immobilized by multipoint covalent attachment were better than the results obtained with the same enzyme in the soluble form decribed by Alquéres et al. [18], which showed around 50% of residual activity after 45 min at 70°C. These results demonstrate that covalent immobilization was fundamental for increasing the thermostability of the biocatalyst. This feature is derived from the larger number of connections between the enzyme and the support, which makes the biocatalyst more rigid.

### 3.3. Optimal Temperature and pH

The effect of pH and temperature on the lipase activity of* P. furiosus* immobilized on a hydrophobic support (octyl-agarose) and a covalent support (glyoxyl-agarose) was studied using factorial design (3^2^). These biocatalysts were chosen because they showed the highest and lowest thermostability. Table 1 shows the experimental conditions (pH and temperature) and the respective lipase activity values for the enzyme immobilized on these supports.

**Figure 2:**
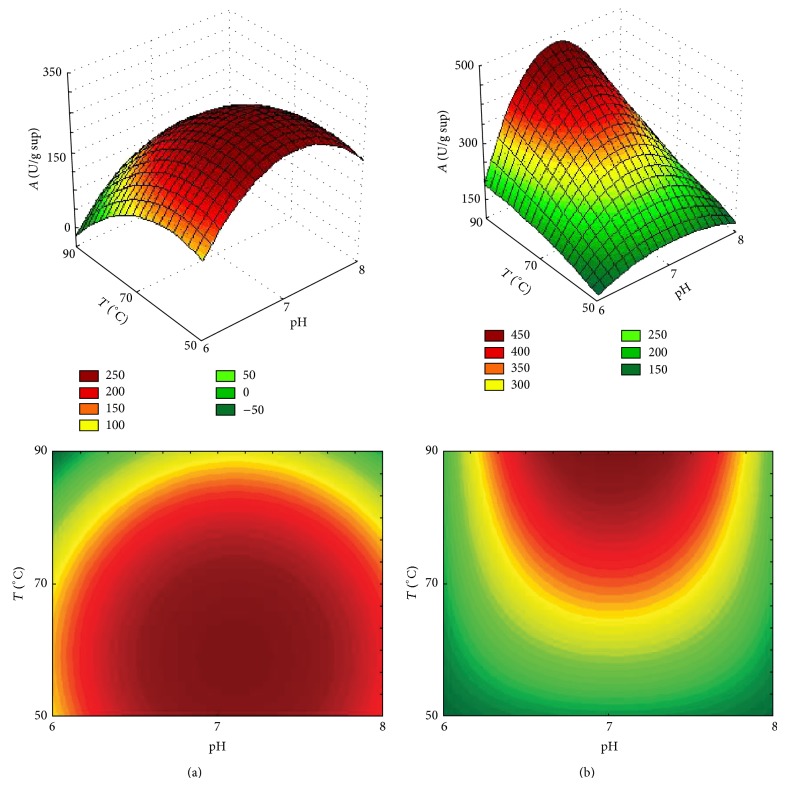
Surface response and contour lines for lipase immobilized on octyl-agarose (a) and on glyoxyl-agarose (b) as a function of temperature and pH. The enzyme immobilized on Octyl-agarose showed an optimal temperature of 66°C and pH around 7. The enzyme immobilized on glyoxyl agarose reached activities at temperatures as high as 90°C and pH 7.

Two models were created: one for octyl-agarose (4) and one for glyoxyl agarose (5). These models describe the activity as a function of temperature and pH of reaction using MUF-Hep as substrate. Response surface graphs were generated from these models (Figures 2(a) and 2(b)):

(4) A=289.61+57.35pH+232.26pH2−108.65T+180.96T2−13.79pH·T+43.56pH·T2−27.29pH2·T+204.96pH2·T2,

(5) A=344.56+8.24pH−166.72pH2+159.38T−23.09T2+3.99pH·T−1.36pH·T2−118.49pH2·T+6.94pH2·T2.

The *F*-test was carried out in order to check the fit of the generated model to the experimental values for the enzyme immobilized on octyl-agarose. It was observed that the calculated *F* (*F*_0.05;8;4_ = 28.16) was higher than the tabulated *F* (8.89), indicating that the model fitted well. The *R* ^2^ value, showing the proximity of the experimental points to the model, was considered very satisfactory, since this value was close to 1 (*R* ^2^ = 0.983). A test was also used to verify the *F*-model's fit to the experimental values generated for the enzyme immobilized on glyoxyl-agarose. It was observed that the calculated *F* (*F*_0.05;8;3_ = 98.78) was higher than the tabulated *F* (19.35), indicating that the model had a good fit. The *R* ^2^ value was considered very satisfactory (*R* ^2^ = 0.996).

According to Figure 2(a), the enzyme immobilized on Octyl-agarose (hydrophobic support) showed an optimal temperature of 66°C and pH around 7. These results are similar to those found by Branco et al. (2010) [17]. When the same enzyme was immobilized on glyoxyl agarose (multipoint covalent support), a wide variation in optimal temperature was found, reaching activities at temperatures as high as 90°C. However, the optimal pH was the same as for the enzyme immobilized on a hydrophobic support. This result shows that through multiple covalent bonds, the enzyme temperature could be increased by approximately 20°C, probably due to the stabilization of the enzyme by the covalent multipoints. The optimal temperature results for the enzyme immobilized by multipoint covalent attachment are higher than the ones found for the same enzyme (soluble) by Alquéres et al. [18], which showed optimal activity at 80°C.

Liu et al. (2009) immobilized a lipase from* Burkholderia *sp. on celite (a support that makes a covalent bond) and carried out a study of temperature and pH. They observed that the immobilized enzyme showed high activity (273.5 U/g) at 55°C and pH 10. Moreover, the authors made a comparison with the soluble enzyme and observed that the optimal pH of the enzyme increased one unit when it was immobilized on celite, although the optimal temperature remained the same [25]. Kuo et al. (2012) immobilized a lipase from* Candida rugosa* on chitosan coated with magnetite, which binds covalently to the support. The authors studied pH ranges and determined that the optimal pH of this biocatalyst was around 7 [26].

Chattopadhyay and Sen (2012) immobilized a pancreatic lipase in two different arrays: egg shells and vegetable fiber. These matrices bind the enzyme in different ways: while the eggshell binds by physical adsorption, the plant fiber binds through covalent bonding. They found that the enzyme immobilized on eggshell showed 7 < pH_optimum_ < 8 while the immobilized enzyme in plant fiber showed 7.5 < pH_optimum_ < 8.5. The optimal temperature for both enzymes was around 35°C. The pH range and optimal temperature for these enzymes were very similar, indicating that pancreatic lipase did not change its properties according to the immobilization method, unlike the results shown in this article [27].

Paula et al. (2008) immobilized a lipase from* Candida rugosa* on a hybrid polysiloxane-polyvinyl support employing different methods, including immobilization by physical adsorption and covalent attachment. They investigated lipase activity in different pH ranges and observed that the enzyme immobilized by physical adsorption showed an optimal pH of 7.5. However, when they employed covalent attachment as the method of immobilization, optimal pH shifted to 8.0 [28]. Paula et al. (2008) also investigated the effect of temperature on these immobilized biocatalysts and observed that the optimal temperature of the fixed systems obtained by physical adsorption (40°C) and covalent attachment (55°C) was higher than the optimal temperature of the free lipase (37°C) [28]. Changes in optimum temperature after immobilization are reported by several authors (Montero et al., 1993, and Fadiloğlu and Söylemez, 1998). However, each system has unique immobilized enzyme characteristics depending on factors such as enzyme source, support type, immobilization method, and enzyme-support interaction [29, 30].

### 3.4. Effect of Immobilization Method on Enzyme Activity and Enantioselectivity

As shown in Table 2, different results were observed dependent of the kind of support and substrate structure. In general, enzymes immobilized by (one-point and multipoint) covalent bonds presented higher activity than enzymes immobilized by hydrophobic adsorption.

Table 2 also shows the effect of a reduction in pH from 7 to 5 when using** 2** and** 4** as substrates. The biocatalyst showed different activity when pH was changed from 7 to 5. When** 4** was used, the decrease in pH (7 to 5) promoted an increase in enzyme activity.

Finally, the enantioselectivity of different immobilized PFUL preparations was evaluated based on the kinetic resolution of (**2**) and (**4**) (Tables 3 and 4). In both cases, the enzyme recognized mainly the R isomer. However, in some cases the enantiomeric ratios *E* were very low (*E* ~ 3) to accurately assess the true enantioselectivity.

The enzyme immobilized on glyoxyl-DTT showed the highest enantioselectivity (*E* = 22). When the pH was changed to 5, the enantiomeric preference was not altered, but the enantiomeric ratios were diminished.

## 4. Conclusions

The results of this work show that a simple immobilization method by covalent biocatalyst improved the characteristics of the enzyme, yielding improved thermostability and higher optimal temperature (*T*_optimum_ = 90°C) than the soluble enzyme (thermostable for 2 h at 75°C and *T*_optimum_ = 70°C), which has been studied previously. Moreover, this new biocatalyst, immobilized by covalent bonds, provided higher enantioselectivity (*E* = 22 and ee (%) = 91) than the biocatalyst immobilized by hydrophobic interaction (*E* = 2 and ee (%) = 35%). These new features offered by immobilization by covalent bonding significantly increase the biotechnological potential of this biocatalyst, expanding its field of use.

## Figures and Tables

**Table 1:** Matrix of the experimental design to determine optimal pH and temperature of the immobilized lipase from *Pyrococcus  furiosus*. Coded and real (in parenthesis) variables and experimental values of enzyme activity for the different experimental conditions.

Assay	pH	Temperature (°C)	Octyl agarose activity (U/g of support)	Glyoxyl agarose activity (U/g of support)
1	−1 (6)	−1 (50)	135.13	117.90
2	0 (7)	−1 (50)	217.30	162.09
3	1 (8)	−1 (50)	190.30	123.68
4	−1 (6)	0 (70)	0	169.60
5	0 (7)	0 (70)	312.05	352.92
6	0 (7)	0 (70)	297.10	352.20
7	0 (7)	0 (70)	244.91	324.40
8	0 (7)	0 (70)	277.44	348.73
9	0 (7)	0 (70)	316.53	—
10	1 (8)	0 (70)	114.70	186.07
11	−1 (6)	1 (90)	0	191.70
12	0 (7)	1 (90)	0	480.85
13	1 (8)	1 (90)	0	213.43

**Table 2:** Activity of different immobilized preparations of *P.  furiosus* lipase in the hydrolysis of different substrates in pH 7.0.

Support	Activity (U/g) in different substrates			
	**1**	**2**	**3**	**4**
	10 mM	5 mM	5 mM	0.5 mM
Octyl-agarose	2982	0.12 (2.39)^*^	3.64	0 (0.03)^*^
Glyoxyl-DTT-agarose	2259	3.08 (0.84)^*^	17.83	0.03 (0.29)^*^
Glyoxyl-agarose	5188	1.01 (0.29)^*^	4.81	0.05 (0.22)^*^

^*^Activity was measured at pH 5.

**Table 3:** Enantioselectivity of different immobilized preparations of *P.  furiosus* lipase in the hydrolysis of **2** at 25°C pH = 7.0.

Supports	ee (%)	*E*	Preference
Octyl-agarose	35 (8.6)^*^	2 (1.2)^*^	S (S)^*^
Glyoxyl-DTT-agarose	91 (46)^*^	22 (2.7)^*^	R (R)^*^
Glyoxyl-agarose	53 (46)^*^	3.2 (2.7)^*^	R (R)^*^

^*^Activity was measured at pH 5.

**Table 4:** Enantioselectivity of different immobilized preparations of *P.  furiosus* lipase in the hydrolysis of **4** at 25°C pH = 7.0.

Supports	ee (%)	*E*	Preference
	pH 7	pH 7	pH 7
Octyl-agarose	65 (21)^*^	4.6 (1.5)^*^	R (R)^*^
Glyoxyl-DTT-agarose	7.4 (61)^*^	1.16 (4.2)^*^	S (R)^*^
Glyoxyl-agarose	27 (52)^*^	1.7 (3.1)	R (R)^*^

^*^Activity was measured at pH 5.
